# Spraying exogenous hormones alleviate impact of weak-light on yield by improving leaf carbon and nitrogen metabolism in fresh waxy maize

**DOI:** 10.3389/fpls.2023.1220827

**Published:** 2023-06-20

**Authors:** Guanghao Li, Wei Li, Yuwen Liang, Weiping Lu, Dalei Lu

**Affiliations:** ^1^Jiangsu Key Laboratory of Crop Genetics and Physiology, Jiangsu Key Laboratory of Crop Cultivation and Physiology, Jiangsu Co-Innovation Center for Modern Production Technology of Grain Crops, Yangzhou University, Yangzhou, China; ^2^Joint International Research Laboratory of Agriculture and Agri-Product Safety, The Ministry of Education of China, Yangzhou University, Yangzhou, China

**Keywords:** weak-light stress, yield, carbon and nitrogen metabolism, fresh waxy maize, exogenous hormones

## Abstract

Insufficient light during the growth periods has become one of the main factors restricting maize yield with global climate change. Exogenous hormones application is a feasible measure to alleviate abiotic stresses on crop productivity. In this study, a field trial was conducted to investigate the effects of spraying exogenous hormones on yield, dry matter (DM) and nitrogen (N) accumulation, leaf carbon and N metabolism of fresh waxy maize under weak-light stress in 2021 and 2022. Five treatments including natural light (CK), weak-light after pollination (Z), spraying water (ZP1), exogenous Phytase Q9 (ZP2) and 6-benzyladenine (ZP3) under weak-light after pollination were set up using two hybrids suyunuo5 (SYN5) and jingkenuo2000 (JKN2000). Results showed that weak-light stress significantly reduced the average fresh ear yield (49.8%), fresh grain yield (47.9%), DM (53.3%) and N accumulation (59.9%), and increased grain moisture content. The net photosynthetic rate (Pn), transpiration rate (Tr) of ear leaf after pollination decreased under Z. Furthermore, weak-light decreased the activities of RuBPCase and PEPCase, nitrate reductase (NR), glutamine synthetase (GS), glutamate synthase (GOGAT), superoxide dismutase (SOD), catalase (CAT) and peroxidase (POD) in ear leaves, and increased malondialdehyde (MDA) accumulation. And the decrease was greater on JKN2000. While ZP2 and ZP3 treatments increased the fresh ear yield (17.8%, 25.3%), fresh grain yield (17.2%, 29.5%), DM (35.8%, 44.6%) and N (42.5%, 52.4%) accumulation, and decreased grain moisture content compared with Z. The Pn, Tr increased under ZP2 and ZP3. Moreover, the ZP2 and ZP3 treatments improved the activities of RuBPCase, PEPCase; NR, GS, GOGAT; SOD, CAT, POD in ear leaves, and decreased MDA content during grain filling stage. The results also showed the mitigative effect of ZP3 was greater than ZP2, and the improvement effect was more significant on JKN2000.

## Introduction

Light provides energy for the generation of plant assimilatory power and acts as a signal for photomorphogenesis (Kumar et al., 2016). Weak-light is a type of abiotic stress that seriously affects plant growth, development and production efficiency. Maize is a typical C_4_ crop, and sufficient light is necessary to ensure its high and stable yield. However, over the past 50 years, global solar radiation has declined at an average rate of 1.4%–2.7% per decade (Stanhill and Cohen, 2001; Ramanathan and Feng, 2009), and the effective sunlight duration declined by 1.28% per decade in China (Che et al., 2005). Studies showed that maize yield reduced by 6%-7% when every 1 MJ/m^2^ decrease in solar radiation (Chen et al., 2020). In southern China, the plum rain season from June and July overlaps with the grain-filling stage of spring maize. However, the grain-filling stage is the key period affecting the yield and quality of waxy maize (Lu et al., 2014). Weak-light stress during grain-filling stage led to yield reduction by reducing grain number and weight (Yang et al., 2016; Shi et al., 2018; Wen et al., 2019), which posed a serious threat to the production safety of maize. Fresh waxy maize is the special maize with the largest planting area in China. The grain starch is almost 100% composed of amylopectin, and the characteristics of high viscosity, low regeneration and easy digestion, made waxy maize the best edible maize. The development of fresh waxy maize plays an important role in promoting the adjustment of planting structure and increasing farmers’ income in China. Therefore, it is of great significance to study how to alleviate the effect of weak-light stress on fresh waxy maize. Weak-light throughout the growth period led to slower growth and development of maize plants, sterility of tassel and ear, lower pollen viability and filament differentiation, and lower DM accumulation, which ultimately resulting in a reduced yield (Cui et al., 2015). Weak-light stress at different periods had different influences on maize, and more yield reduction occurred under weak-light at reproductive growth stage than vegetative growth stage (Yang et al., 2019). Weak-light stress occurred in the early stages has less effect on crop production (Deng et al., 2009), possibly because crop could be able to compensate for stress damage in middle and late stages (Kobata et al., 2000). However, grain number, weight and yield were significantly decreased under weak-light during the grain-filling stage (Deng et al., 2009). Maize grain yield primarily come from direct accumulation of photosynthates at post-silking and remobilization of the non-structural carbohydrate reserved from vegetative organs at pre-silking, and the direct accumulation of photosynthates at post-silking is essential for grain development (Tollenaar and Daynard, 1982; Farooq et al., 2011).

Weak-light stress at post-silking stage decreased chlorophyll content, damaged the ultrastructure of mesophyll cells, decreased photosynthetic capacity (Ren et al., 2016), and reduced DM accumulation, eventually leading to the loss of grain yield (Wang et al., 2020). Previous studies found that weak-light stress after silking significantly reduced the number and size of maize endosperm cells and reduced the enrichment of endosperm cells, which also led to smaller endosperm metastatic cells and the number of mitochondria, which eventually led to lower yield (Jia et al., 2011). Other studies have demonstrated that the IAA, ZR and GA contents in maize grain reduced and the ABA content increased under weak-light stress at post-silking stage, which inhibited grain growth and development (Gao et al., 2018). Studies on other crops also showed that weak-light stress significantly reduced grain yield of wheat (Li et al., 2010) and rice (Wei et al., 2018). N metabolism is an important process for the energy metabolism that determines crop yield and quality. Crop photosynthetic capacity is closely related to leaf N content (Sharwood et al., 2014). RuBPCase accounted for 50 ± 5% of the leaf soluble proteins in C_3_ crop, and it accounted for 10%-25% in C_4_ crop (Schmitt and Edwards, 1981). NR, GS, and GOGAT are the important enzymes involved in the assimilation of intracellular ammonium into organic compounds. Weak-light stress reduced NR, GS, and GOGAT activities in maize leaves, interfered with N metabolism, caused reduction of DM and N accumulation, and ultimately led loss of grain yield (Wang et al., 2020; Sun et al., 2023).

Weak-light stress posed a serious threat to maize production safety. However, there is few effective and reasonable protective measures to abate the influence. In recent years, a wide range of plant growth regulators, such as 6-benzylaminopurine (6-BA), gibberellic acid (GA_3_), auxin (IAA) and cytokinin (CTK) have been widely used to reduce the damage of various abiotic stresses in crop production. The application of IAA and CTK increased grain yield, 1000-grain weight and filled-grain percentage of rice under salt stress (Javid et al., 2011). The application of GA_3_ improved maize growth under salt stress (Rauf et al., 2022). It was well established and known that 6-BA could promote plant cell division, inhibit and scavenge free radicals, delay leaf senescence, increase DM and N accumulation (Roitsch and Ehneß, 2000). Exogenous application of 6-BA effectively alleviated the adverse effects of waterlogging on maize by increasing the leaf area index and chlorophyll content, reducing the MDA content, maintaining the stability of chloroplast ultrastructure (Ren et al., 2017), and reducing the ABA content (Hu et al., 2022). Exogenous 6-BA application during the fertile florets abortion stage of wheat increased the number of florets and number of grains by primarily suppressing the number of degenerated and aborted florets, which result in a further increase in grain yield (Li et al., 2019). Yuan et al. (2014) reported that soaking maize seeds by 6-BA could alleviate the physiological damage under drought stress. At present, most studies on 6-BA regulation of maize growth characteristics focused on waterlogging, drought and other stresses, and few studies reported on regulating growth of fresh waxy maize under weak-light stress. The main component of phytase Q9 is fulvic acid (fulvic acid content≥200 g/L) (Huang et al., 2020). Fulvic acid was reported to have significant effects in stress resistance. Exogenous fulvic acid application substantially reduced the damage of drought stress on maize by sustaining the chlorophyll contents and gas exchange possibly by enhanced SOD, POD and CAT activities and proline levels (Anjum et al., 2011). Huang et al. (2020) found that exogenous spraying phytase Q9 improved the leaf area index, SPAD value and net photosynthetic rate under weak-light conditions at the whole growth period (Huang et al., 2020). However, whether phytase Q9 could alleviate the effect of weak-light stress on fresh waxy maize and its regulation mechanism need further study. This study aimed to investigate the effects of exogenous spraying 6-BA and phytase Q9 on the yield and photosynthetic characteristics of fresh waxy maize under weak-light stress, and provide theoretical basis and technical support for stress-resistant cultivation of fresh waxy maize under climate change.

## Materials and methods

### Experimental design

**Figure 1:**
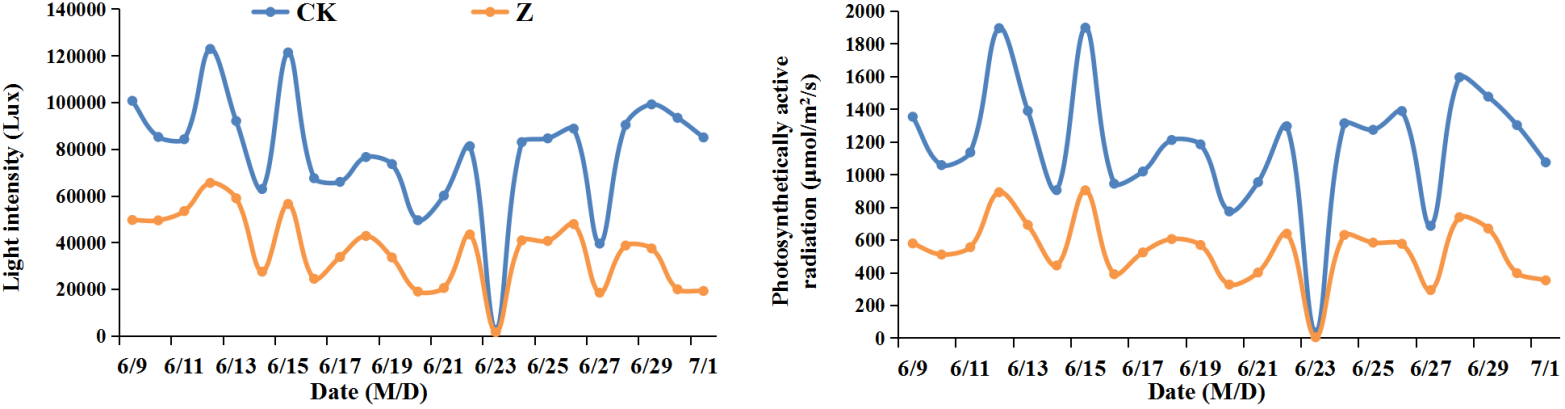
Light intensity and photosynthetically active radiation at daily 10:00 from June 9 to July 1 in 2022. CK, natural light; Z, weak-light after pollination.

The field experiment was conducted at Yangzhou University farm (32°30′N, 119°25′E) in Jiangsu Province, China in the spring maize growing seasons of 2021-2022. Two fresh waxy maize hybrids, Suyunuo5 (SYN5, used in the national fresh waxy maize regional test as the control variety) and Jingkenuo2000 (JKN2000, having the largest planted area of waxy maize in China) were used as experimental materials. The sowing date was March 24 in 2021 and April 4 in 2022, and pollination date was June 14 in 2021 and June 9 in 2022. Maize was planted in double-row (0.8 and 0.4 m) according to local traditional method. Each plot was 10 m × 7.2 m with a plant density of 60000 plants/ha. Slow released compound fertilizer (N/P_2_O_5_/K_2_O=27%/9%/9%) were applied at sowing time with the N/P_2_O_5_/K_2_O rates of 225/75/75 kg/ha. Two hybrids were harvested at milk stage on July 6 and July 1 in 2021 and 2022. After pollination, the shed was built with a black shading net of 50% shading degree (**Figure 1**). The distance between the shading net and the maize canopy was always 2-2.5 m to ensure that the field microclimate under the shading shed is basically consistent with the natural light conditions in the field. Five treatments including natural lighting in the field (CK), shading at 1-23 days after pollination (Z), spraying exogenous water (ZP1), 6-BA (ZP2) and Phytase Q9 (ZP3) under shading at 1-23 days after pollination were set. The concentration of 0.1 g/L 6-BA was used according to previous investigations (Chen et al., 2013). And the concentration of Phytase Q9 was 0.5 g/L. Both exogenous hormones were applied as foliar sprays at the rate of 150 ± 5 mL per plant on all leaves, from 16:00 to 18:00 the next day after shading.

### Yield determination

Thirty ears were harvested from the middle three rows of each plot at milk stage (the 23rd day after pollination) using a continuous sampling method. Based on the average ear weight, three uniform ears were selected from each treatment to determine the fresh grain yield and moisture content.

### Dry matter and N accumulation

Three representative plants of each treatment were collected and separated into leaves and stems (including sheaths and tassels) at silking stage, and into leaves, stems, cobs (including bracts) and grains at milk stage. All samples were oven dried to a constant weight at 80°C after de-enzyming at 105°C for 30 min and weighed separately. After weighing, the samples were ground using a cyclone sample mill with a fine mesh (0.5 mm). N concentrations of different organs were determined using the micro-Kjeldahl method. N accumulations of each fraction were calculated as the product of the concentration and DM.

### Leaf gas exchange parameters

A portable photosynthetic apparatus system (LI-6400 Li-Cor, USA) was used to measure the net photosynthetic rate (Pn), stomatal conductance (Gs), transpiration rate (Tr) and intercellular CO_2_ concentration (Ci) in ear leaves at 10 and 20 days after pollination (DAP). The measurements were performed as described previously (Guo et al., 2023).

### Activities of enzymes involved in carbon, nitrogen metabolism and antioxidant system

At 5, 10, 15 and 21 DAP, Ear leaves of different treatments were collected in liquid N container immediately after sampling for enzyme activities. The activities of RuBPCase, PEPCase, NR, GS, GOGAT, SOD, POD, CAT and the contents of MDA, were measured using MLBIO Plant Sucrose Synthase ELISA Kit, following the manufacturer’s instructions (Shanghai Enzyme-linked Biotechnology Co., Ltd., Shanghai, China) and a previously described method (Saiya-Cork et al., 2002; Zhang et al., 2020).

### Statistical analysis

Statistical calculations were performed in Excel 2016 (Microsoft, Redmond, WA, United States), and figures generated in Sigma Plot 12.0 program. The data were subjected to ANOVA in the General Linear Model module of SPSS. Comparisons among treatments were based on Duncan’s test at the 0.05 probability level (*P*<0.05).

## Results

### Yield

**Figure 2:**
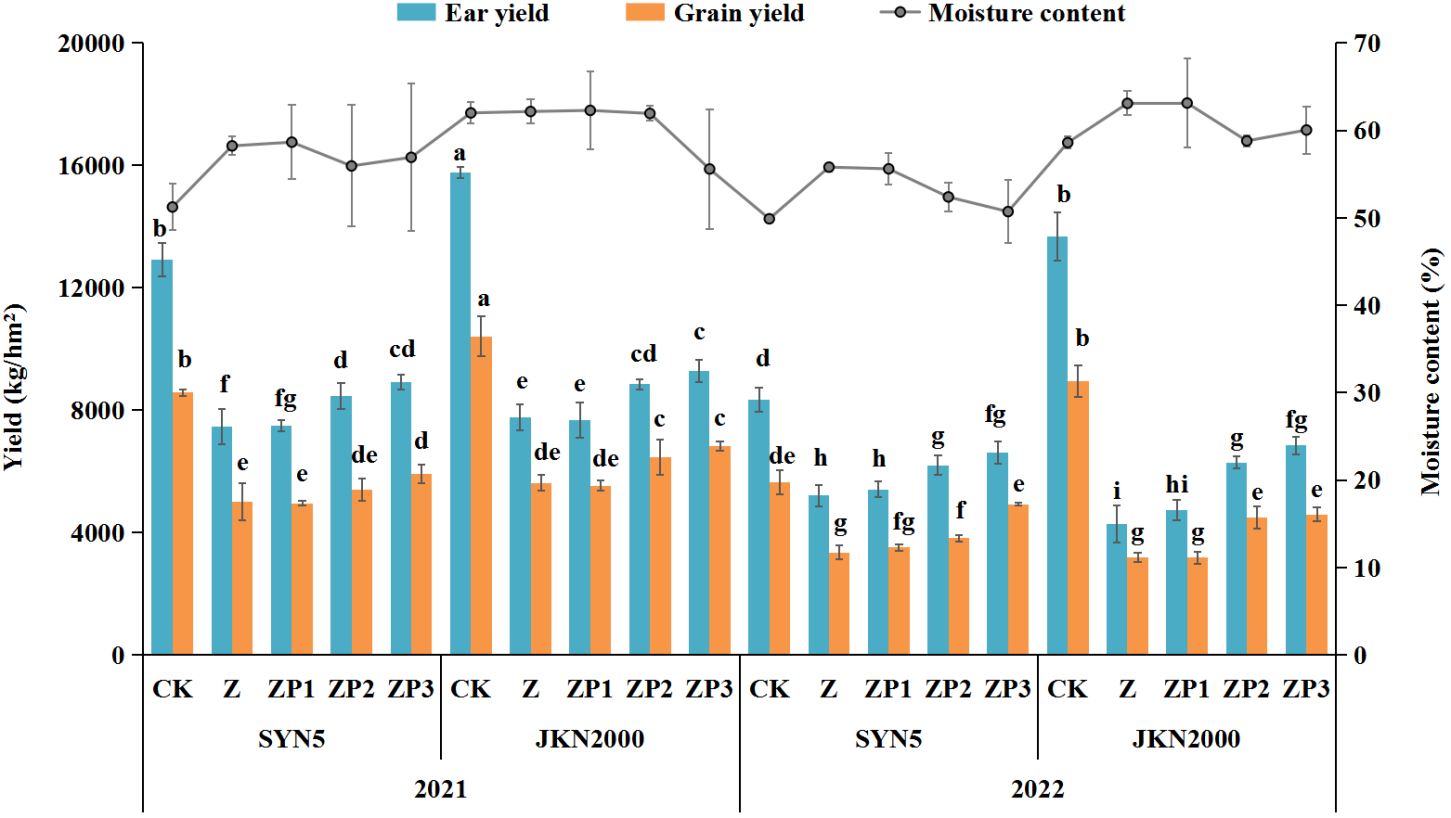
Effects of spraying exogenous hormones on fresh waxy maize yield under weak-light stress. Different letters above the bars represent significant differences at *P* < 0.05. SYN5, Suyunuo5; JKN2000, Jingkenuo2000; CK, natural light; Z, weak-light after pollination; ZP1, ZP2, and ZP3 represent spraying water, Phytase Q9 and 6-BA under weak-light stress after pollination.

**Figure 3:**
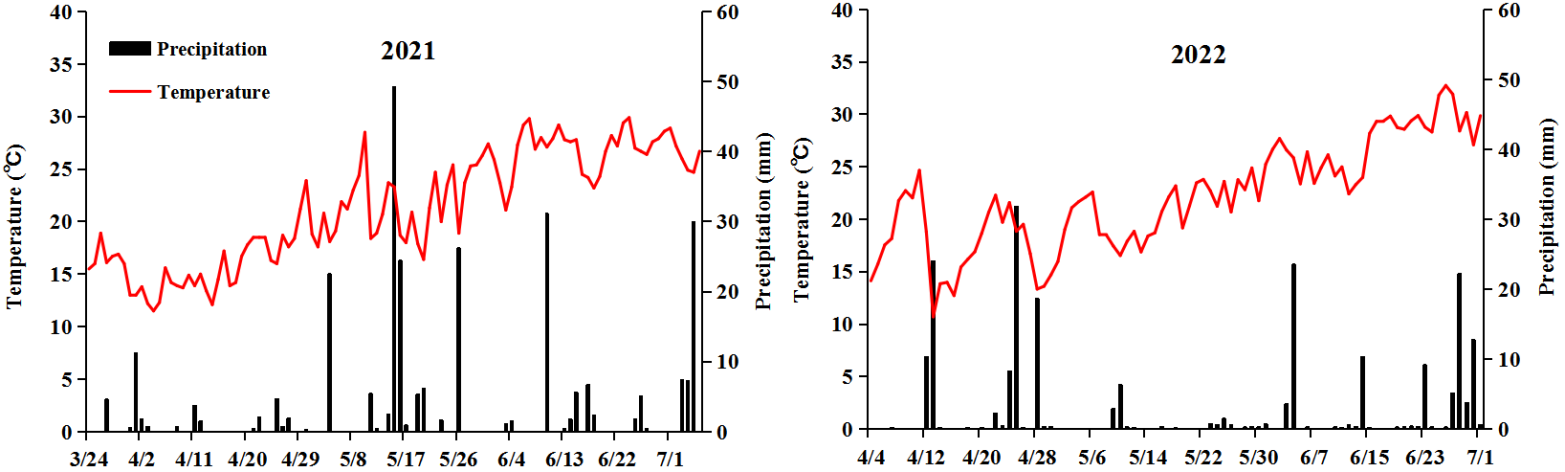
Daily precipitation and average temperature for maize growing season in 2021 and 2022.

Weak-light stress after pollination decreased the fresh ear yield by 40.5% (SYN5) and 59.1% (JKN2000), and decreased by 41.2% and 54.5% in fresh grain yield compared with CK in two years, while increased the moisture content (**Figure 2**). The decrease in JKN2000 was more severe under weak-light stress. Spraying two exogenous hormones increased the fresh ear and grain yield compared with Z, and ZP1 had no significant difference with Z. The fresh ear yields of ZP2 and ZP3 were increased by 13.7% and 20.5%, and the fresh grain yields increased by 8.7% and 27.9% in SYN5. The fresh ear yields of ZP2 and ZP3 were increased by 22.0% and 30.0%, and the fresh grain yields increased by 25.7% and 31.1% in JKN2000. The increase was higher in JKN2000 under ZP3. And spraying exogenous hormones decreased the moisture content in grain to different extents under weak-light. The moisture content of ZP2 and ZP3 were both decreased by 5.8% in SYN5, and by 3.7% and 7.8% in JKN2000. The average yield in 2022 was higher than 2021, perhaps due to the high average temperature in 2022 compared to 2021 (**Figure 3**).

### Dry matter and nitrogen accumulation

**Figure 4:**
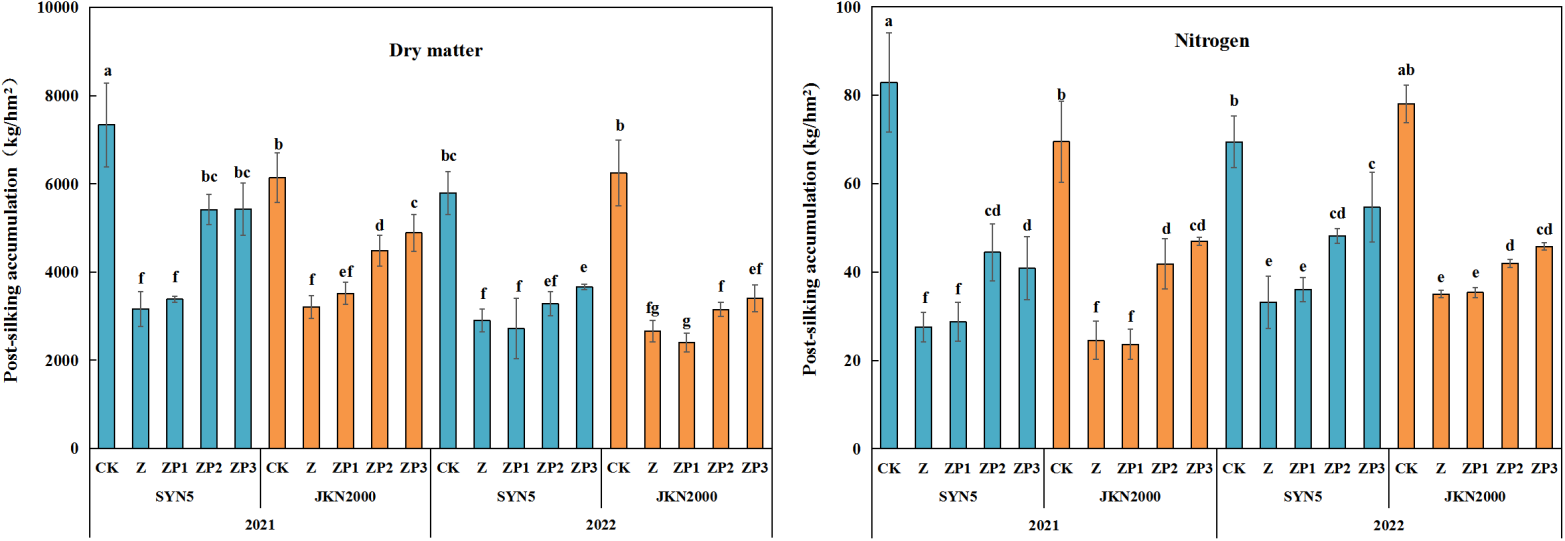
Effects of spraying exogenous hormones on the post-silking accumulation of dry matter and nitrogen in fresh waxy maize under weak-light stress. Different letters above the bars represent significant differences at *P* < 0.05. SYN5, Suyunuo5; JKN2000, Jingkenuo2000; CK, natural light; Z, weak-light after pollination; ZP1, ZP2, and ZP3 represent spraying water, Phytase Q9 and 6-BA under weak-light stress after pollination.

Weak-light stress after pollination significantly reduced DM and N accumulation at post-silking in two years (**Figure 4**). Compared with CK, the DM of Z at post-silking decreased by 53.8% (SYN5) and 52.7% (JKN2000), and the N accumulation of Z were reduced by 60.2% and 59.7%. Compared with Z, the DM of ZP2 and ZP3 at post-silking were increased by 42.5% and 48.9% in SYN5, and by 29.2%, 40.3% in JKN2000. N accumulation of ZP2 and ZP3 at post-silking were increased by 42.9%, 47.4% in SYN5, and by 42.2%, 57.3% in JKN2000. ZP1 had no significant difference with Z. The effect on DM and N under ZP3 was greater than ZP2. The DM and N accumulation in 2022 was higher than 2021.

### Leaf photosynthetic gas exchange parameters

**Figure 5:**
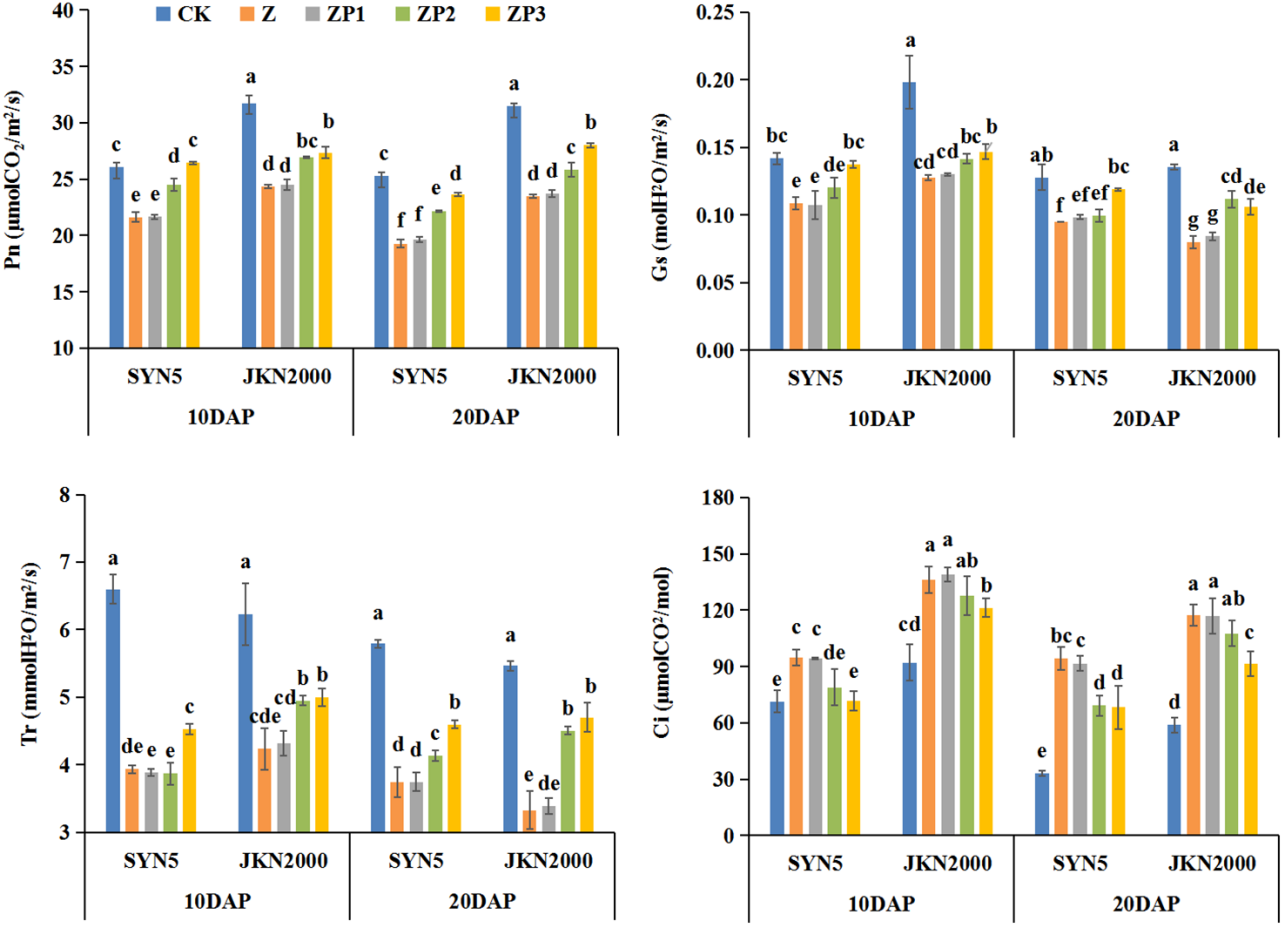
Effects of spraying exogenous hormones on photosynthetic gas exchange parameters in ear leaf of fresh waxy maize under weak-light stress. Different letters above the bars represent significant differences at *P* < 0.05 at same stage. SYN5, Suyunuo5; JKN2000, Jingkenuo2000; CK, natural light; Z, weak-light after pollination; ZP1, ZP2, and ZP3 represent spraying water, Phytase Q9 and 6-BA under weak-light stress after pollination. Pn, photosynthetic rate; Gs, stomatal conductance; Tr, transpiration rate; Ci, intercellular CO_2_.

Pn, Tr, Gs and Ci were higher at 10DAP than those at 20DAP. Compared with CK, Z treatment decreased Pn, Tr, and Gs, but increased Ci at 10DAP and 20DAP (**Figure 5**). ZP2 and ZP3 treatments increased the Pn of SYN5 and JKN2000 compared with Z, and the increase was higher at ZP3. The GS of ZP3 were increased significantly, but it had no significance with that of ZP2. The Tr of ZP2 and ZP3 were increased compared with Z. The Ci of two cultivars were decreased under ZP3 treatment, and ZP2 decreased the Ci in SYN5, but it had no significant effect on JKN2000. Overall, 6-BA had a more impact on the leaf photosynthetic gas exchange parameters.

### Activities of enzymes involved in carbon and nitrogen metabolism

**Figure 6:**
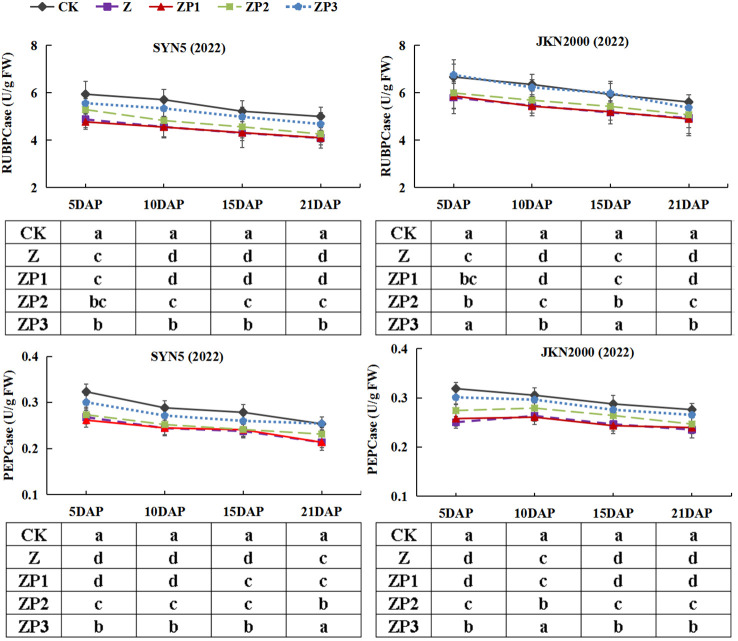
Effects of spraying exogenous hormones on the activities of RuBPCase and PEPCase in ear leaf of fresh waxy maize under weak-light stress. SYN5, Suyunuo5; JKN2000, Jingkenuo2000; CK, natural light; Z, weak-light after pollination; ZP1, ZP2, and ZP3 represent spraying water, Phytase Q9 and 6-BA under weak-light stress after pollination. RuBPCase, ribulose-1,5-bisphosphate carboxylase; PEPase, phosphoenolpyruvate carboxylase.

The activities of RuBPCase and PEPCase gradually decreased after pollination in two hybrids in 2022 (**Figure 6**). RuBPCase and PEPCase activities were decreased under Z treatment at all stages. Compared with Z, ZP3 treatment increased the RuBPCase and PEPCase activities significantly. The RuBPCase activities in two hybrids and PEPCase activity in JKN2000 were increased under ZP2. ZP1 have no significant effect on activities of RuBPCase and PEPCase. In general, compared with Z, the average RuBPCase activities of the two hybrids under ZP2 and ZP3 were increased 6.6% and 15.9% in SYN5, 3.7% and 13.6% in JKN2000. And the average PEPCase activities of the two hybrids under ZP2 and ZP3 were increased 4.0% and 13.2% in SYN5, 6.2% and 13.6% in JKN2000.

**Figure 7:**
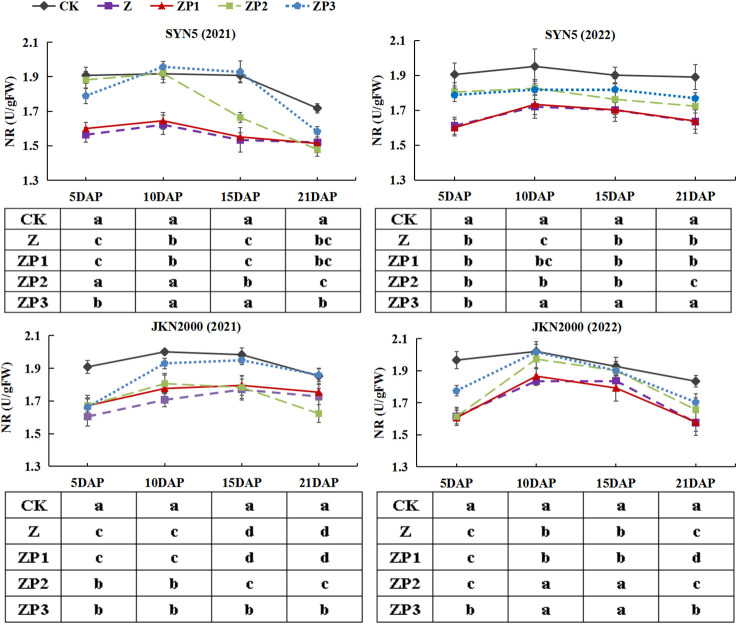
Effects of spraying exogenous hormones on the activities of NR in ear leaf of fresh waxy maize under weak-light stress. SYN5, Suyunuo5; JKN2000, Jingkenuo2000; CK, natural light; Z, weak-light after pollination; ZP1, ZP2, and ZP3 represent spraying water, Phytase Q9 and 6-BA under weak-light stress after pollination. NR, nitrate reductase.

**Figure 8:**
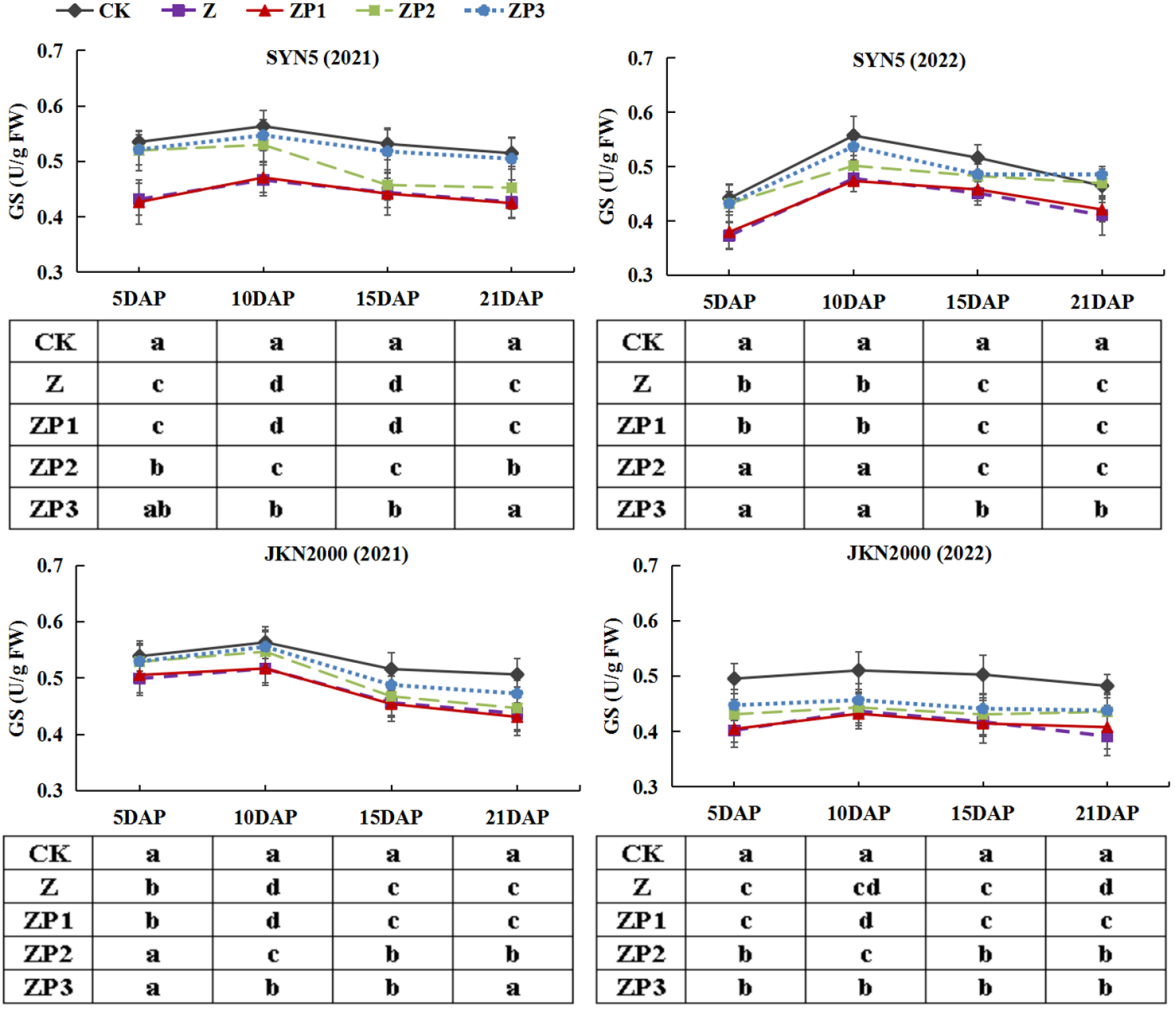
Effects of spraying exogenous hormones on the activities of GS in ear leaf of fresh waxy maize under weak-light stress. SYN5, Suyunuo5; JKN2000, Jingkenuo2000; CK, natural light; Z, weak-light after pollination; ZP1, ZP2, and ZP3 represent spraying water, Phytase Q9 and 6-BA under weak-light stress after pollination. GS, glutamine synthetase.

**Figure 9:**
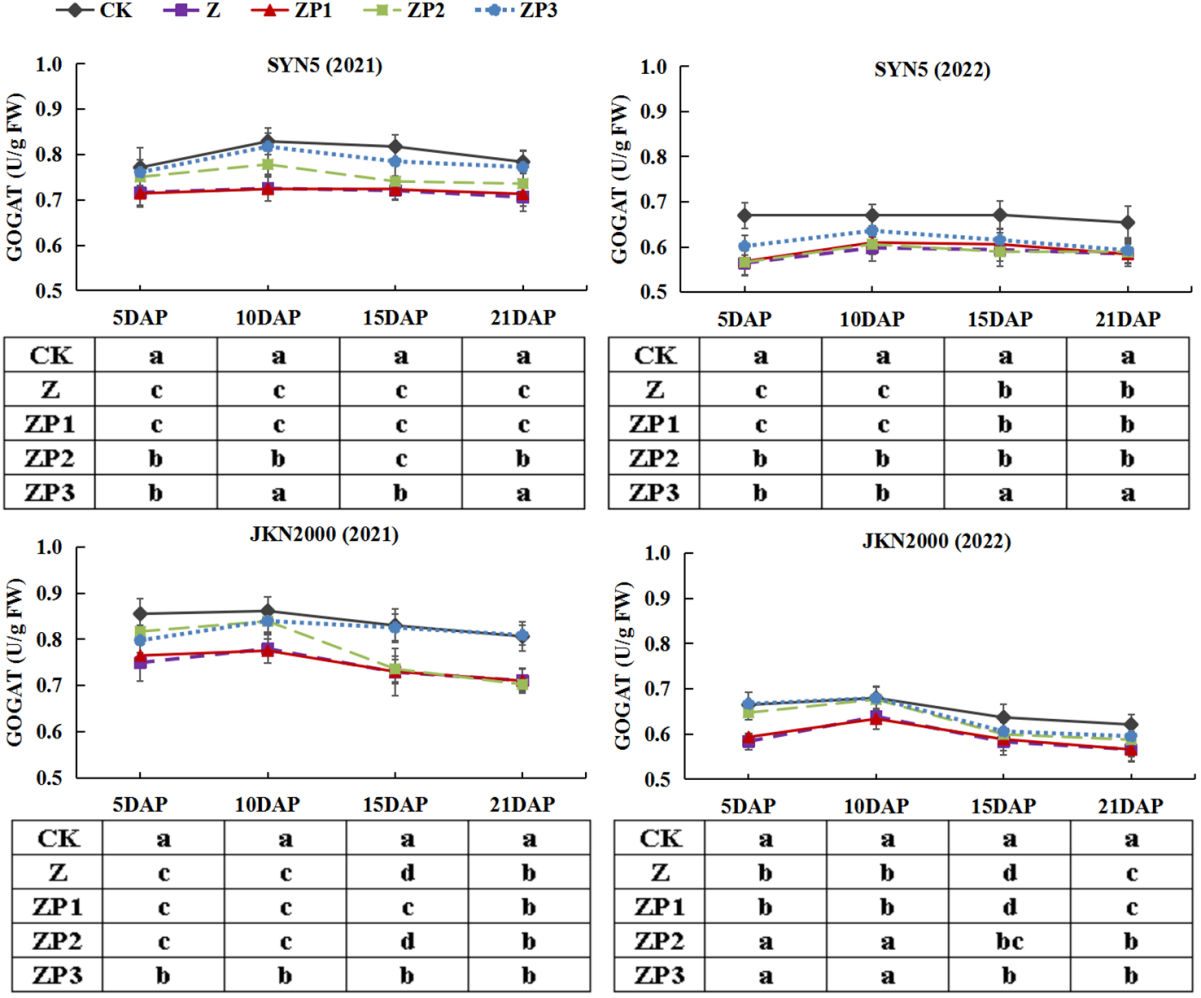
Effects of spraying exogenous hormones on the activities of GOGAT in ear leaf of fresh waxy maize under weak-light stress. SYN5, Suyunuo5; JKN2000, Jingkenuo2000; CK, natural light; Z, weak-light after pollination; ZP1, ZP2, and ZP3 represent spraying water, Phytase Q9 and 6-BA under weak-light stress after pollination. GOGAT, glutamate synthase.

NR, GS and GOGAT activities of the two hybrids increased initially, peaked at 10 DAP and decreased afterwards in two years (**Figures 7**–**9**). Weak-light stress decreased the activities of NR, GS and GOGAT after pollination in both hybrids. ZP2 and ZP3 treatments increased the activities of NR, GS and GOGAT compared with Z. And the increase was greater under ZP3. Compared with Z, the NR activities of SYN5 and JKN2000 were increased by 8.3% and 1.3% under ZP2 and increased by 11.4% and 6.9% under ZP3. The GS activities of SYN5 and JKN2000 were increased by 10.1% and 4.6% under ZP2 and increased by 15.4% and 7.4% under ZP3. And the activities of GOGAT in SYN5 and JKN2000 were increased by 1.9% and 4.5% under ZP2 and increased by 6.2% and 8.4% under ZP3. The trend of NR and GS activities were consistent between two hybrids and between two years.

### Activities of antioxidant enzymes and MDA content

**Figure 10:**
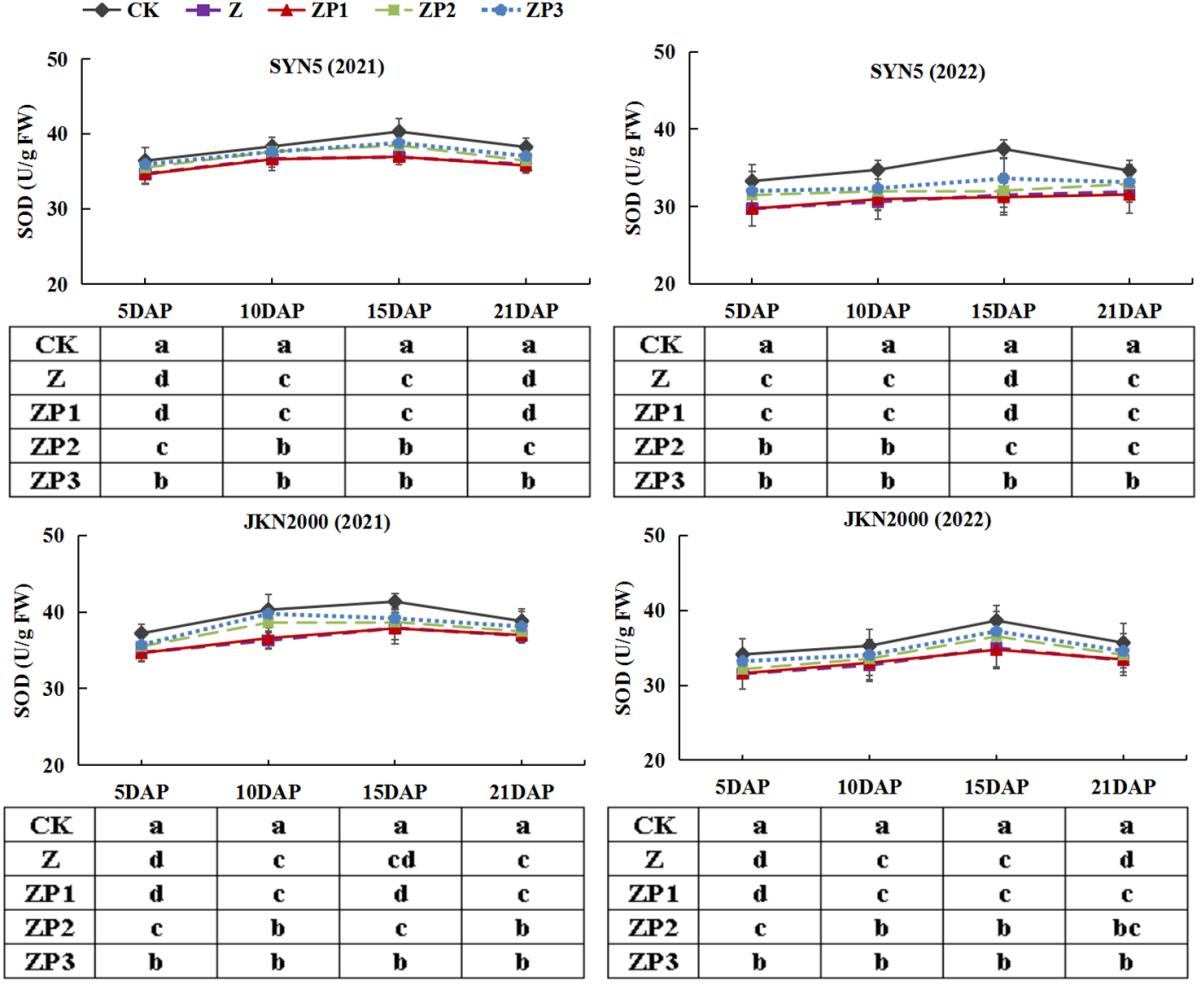
Effects of spraying exogenous hormones on the activities of SOD in ear leaf of fresh waxy maize under weak-light stress. SYN5, Suyunuo5; JKN2000, Jingkenuo2000; CK, natural light; Z, weak-light after pollination; ZP1, ZP2, and ZP3 represent spraying water, Phytase Q9 and 6-BA under weak-light stress after pollination. SOD, superoxide dismutase.

**Figure 11:**
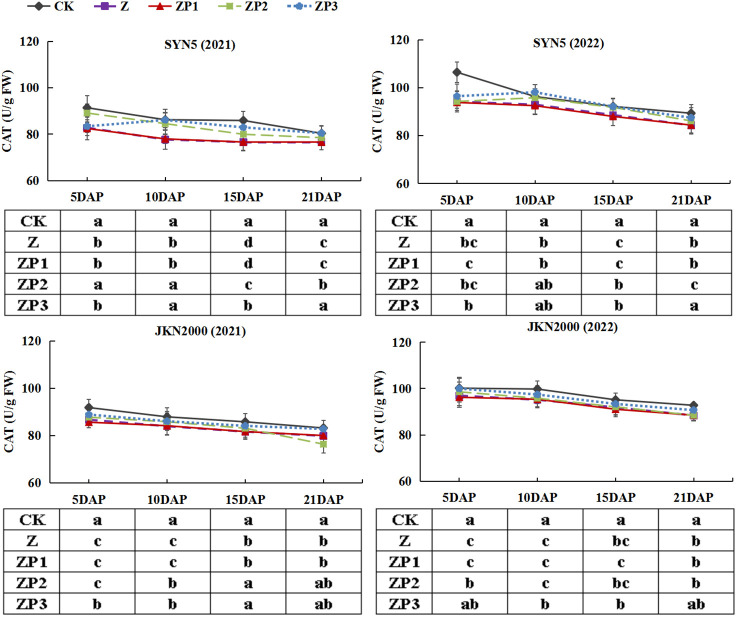
Effects of spraying exogenous hormones on the activities of CAT in ear leaf of fresh waxy maize under weak-light stress. SYN5, Suyunuo5; JKN2000, Jingkenuo2000; CK, natural light; Z, weak-light after pollination; ZP1, ZP2, and ZP3 represent spraying water, Phytase Q9 and 6-BA under weak-light stress after pollination. CAT, catalase.

**Figure 12:**
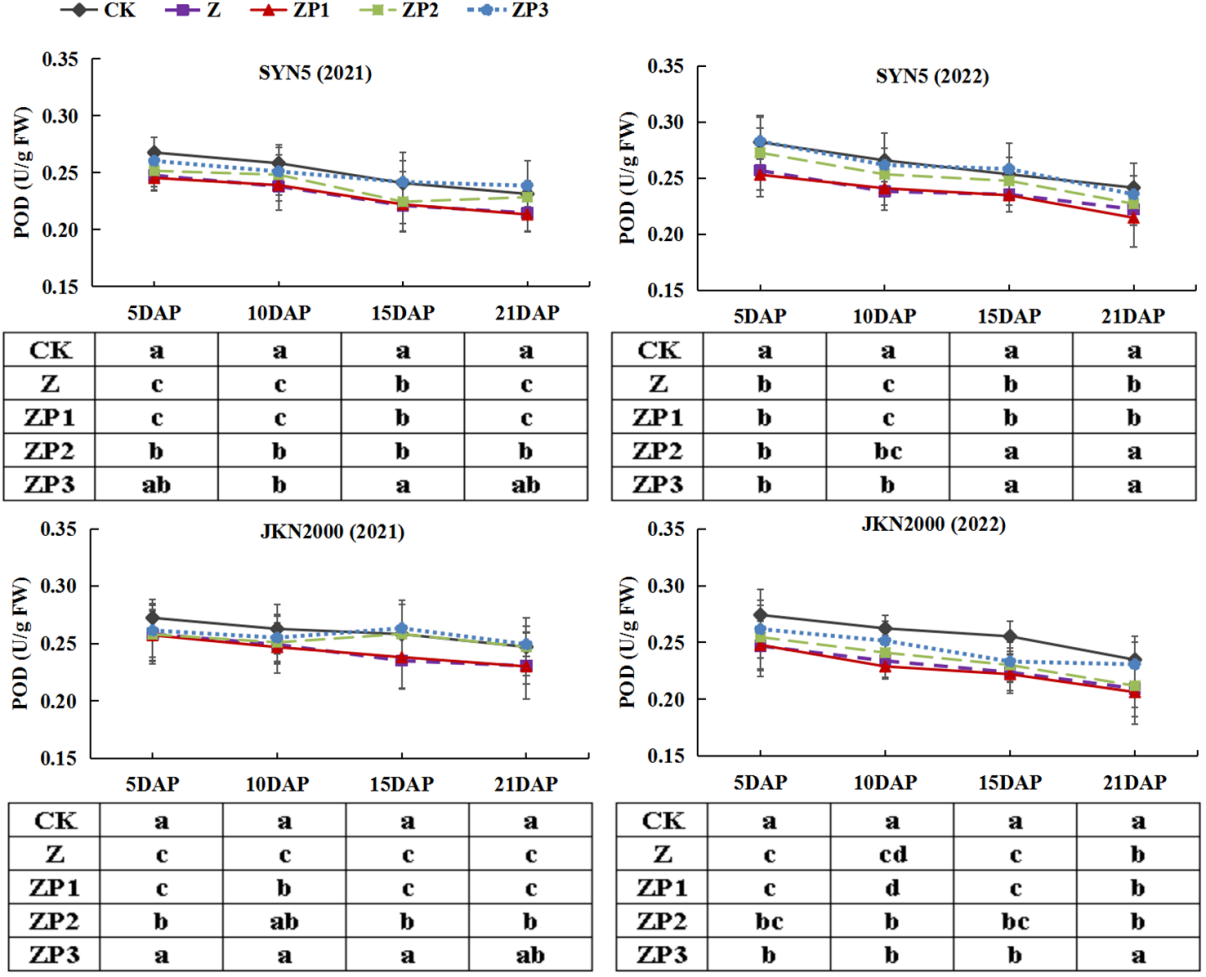
Effects of spraying exogenous hormones on the activities of POD in ear leaf of fresh waxy maize under weak-light stress. SYN5, Suyunuo5; JKN2000, Jingkenuo2000; CK, natural light; Z, weak-light after pollination; ZP1, ZP2, and ZP3 represent spraying water, Phytase Q9 and 6-BA under weak-light stress after pollination. POD, peroxidase.

**Figure 13:**
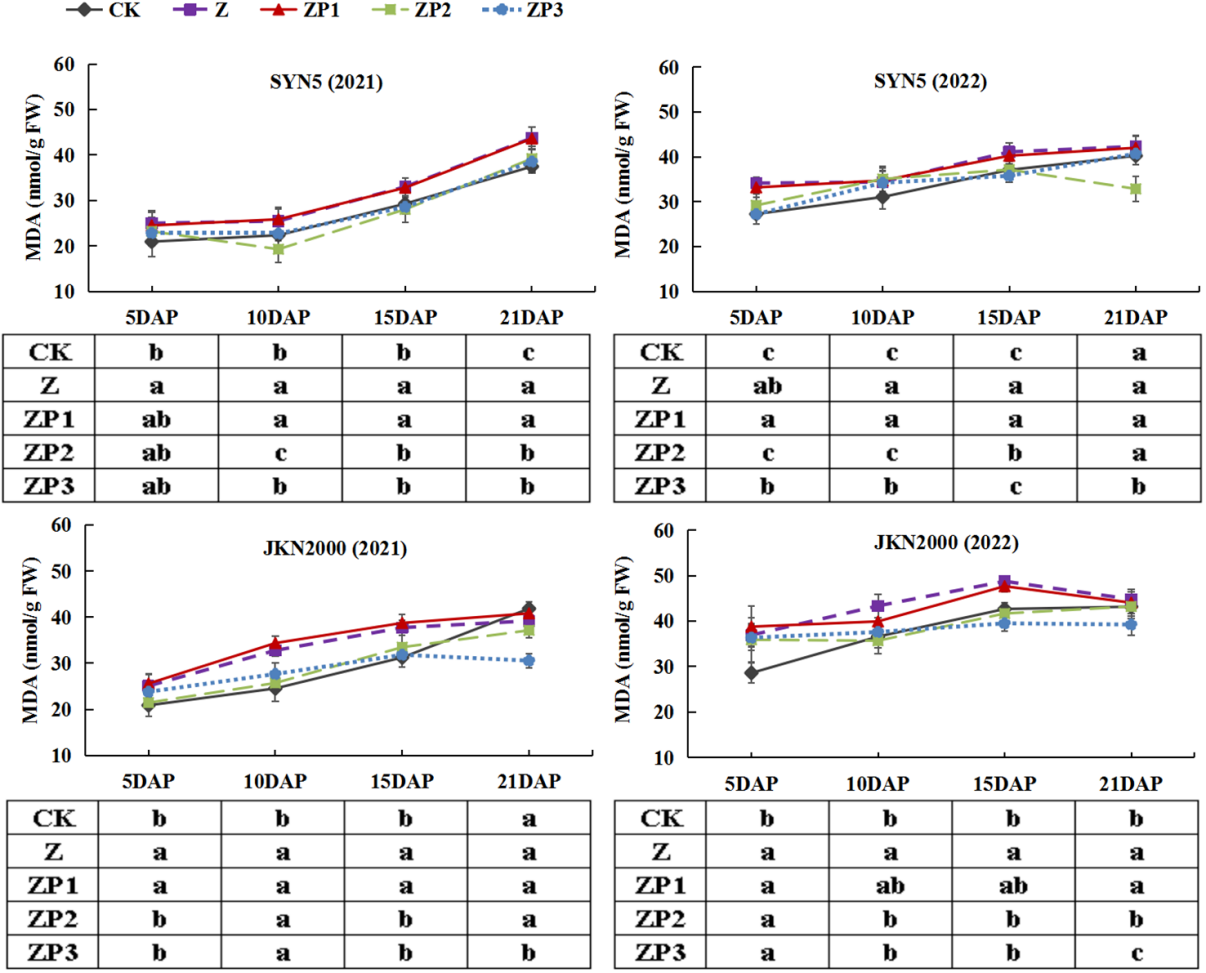
Effects of spraying exogenous hormones on MDA content in ear leaf of fresh waxy maize under weak-light stress. SYN5, Suyunuo5; JKN2000, Jingkenuo2000; CK, natural light; Z, weak-light after pollination; ZP1, ZP2, and ZP3 represent spraying water, Phytase Q9 and 6-BA under weak-light stress after pollination. MDA, malondialdehyde.

The SOD activities of the two hybrids increased initially, peaked at 15DAP and decreased afterwards in two years. Whereas the activities of CAT and POD gradually decreased with grain growth (**Figures 10**–**12**). The activities of SOD, CAT and POD were decreased under Z. ZP2 and ZP3 increased the activities of SOD, CAT and POD, but the increase was dependent on hybrid, stage and year. Compared with Z, the SOD activities of SYN5 and JKN2000 were increased by 3.4% and 2.7% under ZP2, and increased by 5.0% and 4.6% under ZP3. The CAT activities of SYN5 and JKN2000 were increased by 4.2% and 0.7% under ZP2, and increased by 5.3% and 2.9% under ZP3. POD activities of SYN5 and JKN2000 were increased by 4.9% and 4.0% under ZP2, and increased by 9.0% and 7.0% under ZP3. Meanwhile, the content of MDA gradually increased with grain growth, and it was increased under Z (**Figure 13**). But under ZP2 and ZP3, the contents of MDA decreased compared with Z. The content of MDA in SYN5 and JKN2000 were decreased by 12.0% and 12.1% under ZP2, and decreased by 9.6% and 13.7% under ZP3. The trend of SOD and POD activities were consistent between two hybrids and between two years.

### Correlation analysis

**Figure 14:**
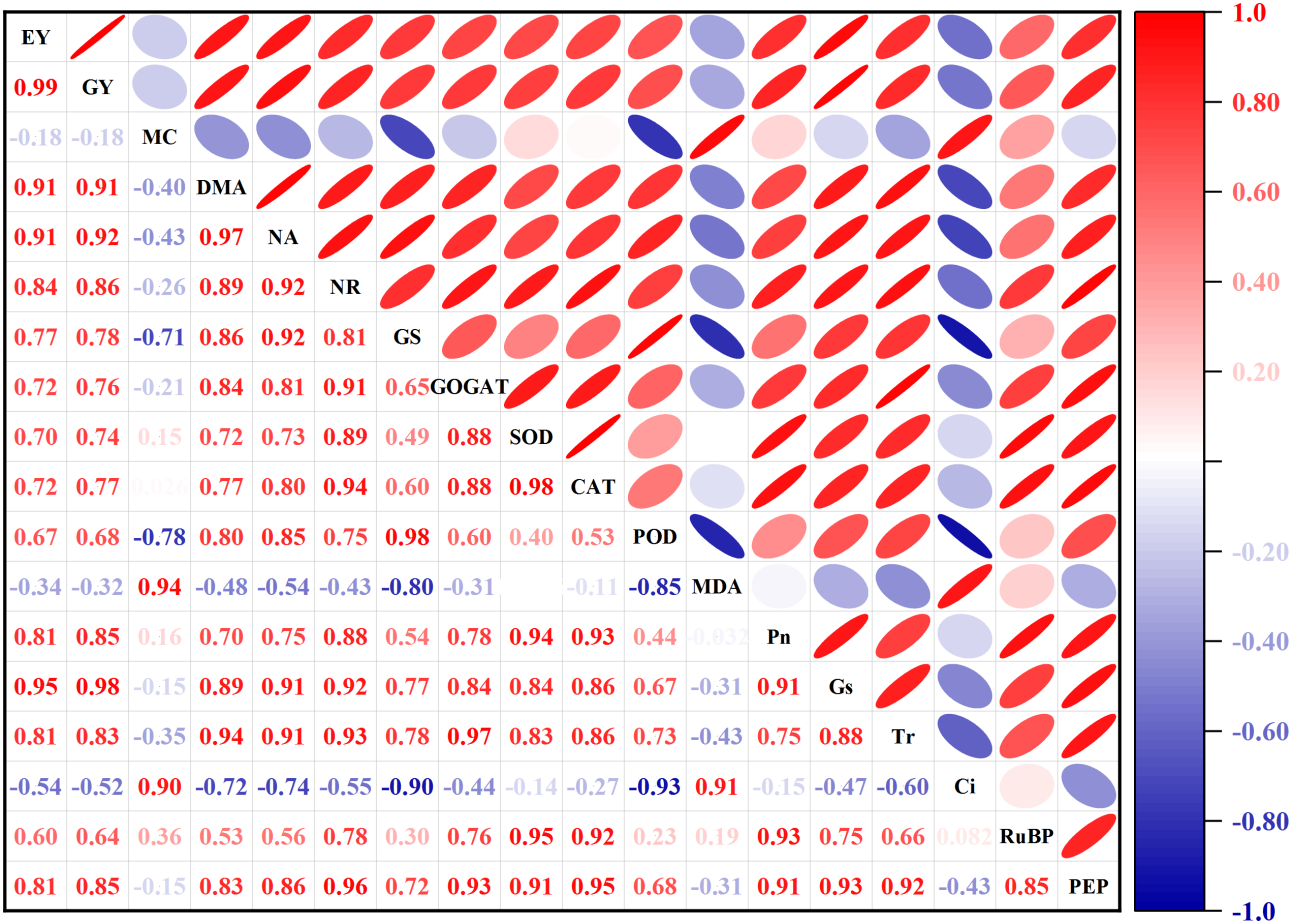
Pearson correlation matrix between yield, dry matter and N accumulation after pollination, and leaf related parameters. EY, fresh ear yield; GY, fresh grain yield; MC, moisture content; DMA, dry matter accumulation; NA, nitrogen accumulation; NR, nitrate reductase; GS, glutamine synthetase; GOGAT, glutamate synthase; SOD, superoxide dismutase; CAT, catalase; POD, peroxidase; MDA, malondialdehyde; Pn, photosynthetic rate; Gs, stomatal conductance; Tr, transpiration rate; Ci, intercellular CO_2_; RuBP, ribulose-1,5-bisphosphate carboxylase; PEP, phosphoenolpyruvate carboxylase.

In this study, Pearson’s correlation analysis revealed fresh ear and grain yield had positive correlations with DM and N accumulation, N metabolism enzymes (NR, GS, GOGAT), antioxidant enzymes (SOD, CAT, POD), photosynthetic gas exchange parameters (Pn, Tr, Gs), and carbon metabolism enzymes (RuBPCase, PEPCase) (**Figure 14**). The DM and N accumulation were positively correlated with carbon and N metabolism enzymes, antioxidant enzymes and photosynthetic gas exchange parameters. The carbon and N metabolism enzymes had positive correlations with antioxidant enzymes and photosynthetic gas exchange parameters. And the antioxidant enzymes had positive correlations with photosynthetic gas exchange parameters. The correlation analysis also indicated that photosynthetic gas exchange parameters were positively correlated with RuBPCase and PEPCase. Ci and MDA were negatively correlated with other indexes or parameters.

## Discussion

Solar radiation is the energy source for the accumulation of photosynthates (Yang et al., 2021a). Photosynthate accumulation especially at post-silking is a key determinant of maize yield (Barnabás et al., 2008; Liu et al., 2020). The leaf N content is closely associated with plant photosynthetic capacity. A high N content in leaves enhanced photosynthesis and delayed leaf senescence (Sinclair et al., 2000). In this study, the results showed that weak-light stress decreased fresh ear and grain yield, DM and N accumulation in fresh waxy maize, which was consistent with the previous results (Yang et al., 2021a; Yang et al., 2021b). Exogenous hormone played an important role in the response to various abiotic stresses. Previous studies have showed that spraying phytase Q9 improved yield and DM accumulation on summer maize (Huang et al., 2020), and this is consistent with the results in this study on fresh waxy maize. Furthermore, we also found that spraying 6-BA and phytase Q9 both increased the yield, DM and N accumulation, and the increase was greater in 6-BA.

Photosynthetic capacity is the major determinant of crop productivity. Our study showed that weak-light stress decreased the Pn of ear leaves, which was consistent with the previous results on normal maize (Sinclair et al., 2000; Sharwood et al., 2014). Weak-light stress reduced leaf area index (LAI) and chlorophyll content and damaged the mesophyll cell ultrastructure, which led to the decrease of photosynthetic capacity, and thus resulted in significant yield loss (Ren et al., 2016). Previous study found that LAI, SPAD value and Pn of summer maize significantly increased under spraying phytase Q9 (Huang et al., 2020). In this study, we found that spraying 6-BA and phytase Q9 increased Pn significantly under weak-light stress, and the increase was higher in 6-BA. RuBPCase and PEPCase are key enzymes involved in carbon metabolism, and NR, GS, GOGAT are key enzymes involved in N metabolism. The activities of RuBPCase, PEPCase, NR, GS and GOGAT were decreased under weak-light on summer maize (Sharwood et al., 2014; Wang et al., 2020). This is generally consistent with the results of this experiment in fresh waxy maize. Studies reported that weak-light stress reduced the activities of photosynthesis-related enzymes in maize leaves (Sharwood et al., 2014), which reduced the net photosynthetic rate and led to the decrease of photosynthetic production capacity (Zhong et al., 2014). The decrease in grain weight and volume under weak-light may be due to reduced photosynthetic rate and decreased assimilate production capacity resulting from the inhibition of leaf N metabolism (Sharwood et al., 2014). In this study, spraying 6-BA and phytase Q9 increased the activities of PEPCase, RuBPCase, NR, GS and GOGAT under weak-light stress. This promoted the improvement of photosynthetic capacity, which in turn increased DM and N accumulation. Our experimental results also indicated that the spraying effect on carbon and N metabolism of 6-BA was better.

SOD, CAT and POD are key enzymes involved in antioxidant systems, and MDA is one of the products of membrane lipid peroxidation, its content can be used as one of the indicators to investigate the severity of cell stress. Previous study found that weak-light stress reduced the activities of SOD, CAT and POD, and increased the MDA content in ear leaves of summer maize (Huang et al., 2020). In this study, we also found that weak-light stress decreased the activities of SOD, CAT and POD, and increased the content of MDA, which accelerated the oxidative damage of leaf in fresh waxy maize. Under weak-light stress, plasmolysis occurred in mesophyll cells and the endomembrane system was destroyed, which resulted in the dissolution of cell membrane, karyotheca, mitochondria, and the mesophyll cell ultrastructure was damaged, which led to the decrease of photosynthetic capacity, and thus resulted in yield loss (Ren et al., 2016). Spraying phytase Q9 could increased the activities of SOD, CAT and POD (Huang et al., 2020). Our results also indicated that spraying 6-BA and phytase Q9 increased the activities of SOD, CAT and POD, and reduced the MDA content, which delayed the leaf senescence. We also found that the spraying effect on antioxidant systems of 6-BA was better than phytase Q9. Spraying exogenous hormones significantly alleviated the decline in anti-aging ability and photosynthetic productivity of fresh waxy maize under weak-light stress, thereby promoting dry matter accumulation and increasing the fresh ear and grain yield. Further research is needed on the hormone metabolism and molecular physiological mechanisms of exogenous regulators applied to improve the yield of fresh waxy maize under light stress.

## Conclusions

The results showed that weak-light stress during the grain-filling stage severely limited photosynthetic production capacity by reducing the activity of enzymes involved in carbon and N metabolism, accelerating leaf senescence, and thereby reducing the accumulation of photosynthetic products, resulting in reduced yield in fresh waxy maize. The effect of weak-light stress was greater on JKN2000 than SYN5. Spraying exogenous phytase Q9 and 6-BA alleviated the decrease in carbon and nitrogen metabolism enzymes and antioxidant enzyme activities under weak-light stress, improved photosynthetic capacity, delayed leaf senescence, and thus increased dry matter and nitrogen accumulation, increasing yield. The improvement effect of 6-BA was more significant on JKN2000. These results indicated that in the actual production of fresh waxy maize, 6-BA can be widely used as an effective regulator to alleviate weak-light stress.

## Data availability statement

The raw data supporting the conclusions of this article will be made available by the authors, without undue reservation.

## Author contributions

GL, WL, and DL designed the experiments. GL,WL, and YL did the field management. GL, WL, and WPL performed data analysis. GL, WL, and DL contributed to the preparation of the manuscript. All authors contributed to the article and approved the submitted version
